# FDXR drives primary and endocrine-resistant tumor cell growth in ER+ breast cancer via CPT1A-mediated fatty acid oxidation

**DOI:** 10.3389/fonc.2023.1105117

**Published:** 2023-05-03

**Authors:** Chaojun Yan, Ronghui Gao, Chuan Gao, Kai Hong, Meng Cheng, Xiaojing Liu, Qing Zhang, Jing Zhang

**Affiliations:** ^1^ Department of Thyroid and Breast Surgery, Medical Research Institute, Frontier Science Center for Immunology and Metabolism, Zhongnan Hospital of Wuhan University, Wuhan University, Wuhan, China; ^2^ Department of Medical Ultrasound, Tongji Hospital, Tongji Medical College, Huazhong University of Science and Technology, Wuhan, China; ^3^ Lineberger Comprehensive Cancer Center, University of North Carolina School of Medicine, Chapel Hill, NC, United States; ^4^ Department of Pharmacology and Cancer Biology, Duke University School of Medicine, Durham, NC, United States; ^5^ Department of Pathology, University of Texas Southwestern Medical Center, Dallas, TX, United States; ^6^ Simmons Comprehensive Cancer Center, University of Texas Southwestern Medical Center, Dallas, TX, United States

**Keywords:** breast cancer, endocrine resistance, ferredoxin reductase, palmitoyltransferase 1A, fatty acid oxidation, combination therapy

## Abstract

**Background:**

The majority of breast cancers (BCs) expressing estrogen receptor (ER) have shown endocrine resistance. Our previous study demonstrated that ferredoxin reductase (FDXR) promoted mitochondrial function and ER+ breast tumorigenesis. But the underlying mechanism is not clear.

**Methods:**

Liquid chromatography (LC) tandem mass spectrometry (MS/MS)-based metabolite profiling was utilized to reveal the metabolites regulated by FDXR. RNA microarray was utilized to determine the potential downstream targets of FDXR. Seahorse XF24 analyzer was performed to analyze the FAO-mediated oxygen consumption rate (OCR). Q-PCR and western blotting assays were used to measure expression levels of FDXR and CPT1A. MTS, 2D colony formation and anchorage-independent growth assays were used to evaluate the effects of FDXR or drug treatments on tumor cell growth of primary or endocrine-resistant breast cancer cells.

**Results:**

We found that depletion of FDXR inhibited fatty acid oxidation (FAO) by suppressing CPT1A expression. Endocrine treatment increased the expression levels of both FDXR and CPT1A. Further, we showed that depletion of FDXR or FAO inhibitor etomoxir treatment reduced primary and endocrine-resistant breast cancer cell growth. Therapeutically, combining endocrine therapy with FAO inhibitor etomoxir synergistically inhibits primary and endocrine-resistant breast cancer cell growth.

**Discussion:**

We reveal that the FDXR-CPT1A-FAO signaling axis is essential for primary and endocrine-resistant breast cancer cell growth, thus providing a potential combinatory therapy against endocrine resistance in ER+ breast cancer.

## Introduction

Breast cancer is the leading cause of cancer death among women. Approximately 75% of breast cancer is estrogen receptor-positive (ER+), which leads to the majority of breast cancer deaths (1–3). Despite treatment with antiestrogen therapy, up to 50% of patients with ER+ breast cancer do not benefit from these treatments due to intrinsic or acquired resistance (4–9). Recently, scientists have elucidated altered molecular signal transduction pathways and genetic driver mutations involved in the development of endocrine resistance, thereby identifying novel therapeutic targets to improve patient outcomes, such as mTOR inhibitors or cyclin-dependent kinase (CDK) 4/6 inhibitors; however, there are also similar problems with intrinsic or acquired resistance in patients (10–19), highlighting the urgent need for additional effective therapies.

Altered metabolism, which tumorigenesis heavily depends on to support uncontrolled cell proliferation, is a hallmark of cancer (20, 21). Cancer metabolic programs include the reprogramming of glycolysis, glutaminolysis, oxidative phosphorylation, fatty acid metabolism, and one-carbon metabolism, which provide essential energy, biosynthesis factors and intermediates for tumor growth, division and redox homeostasis (22). Therefore, tumor cell metabolism has been considered the Achilles’ heel of cancer and is a successful therapeutic target (23–25). For example, the mitochondrial complex I inhibitor metformin is approved for the treatment of type 2 diabetes and has been reported to possess anticancer activity in various investigations and clinical trials (26–30). The fatty acid oxidation inhibitor perhexiline is approved to treat angina and exhibits anticancer effects *in vitro* and *in vivo* (31–33). Although targeting cancer metabolic alterations holds promise, identifying novel predictive biomarkers is needed to lead to precision therapy (23).

Emerging evidence has shown that tumor cells derive most of their ATP from mitochondria-mediated oxidative phosphorylation that is mainly driven by glucose metabolism, glutaminolysis and fatty acid oxidation (34–38). Recent evidence has demonstrated that mitochondrial oxidative metabolism drives therapeutic resistance, suggesting an important role of mitochondrial inhibitors in preventing cancer progression (39, 40). ER+ breast cancer cells depend more on mitochondrial function to provide the essential ATP needed for survival than other subtypes of breast cancer (35, 41). Therefore, identifying the mechanisms that control mitochondrial function in ER+ breast cancer will potentially lead to the development of novel therapeutic interventions. Research from our group and others has demonstrated that the proline hydroxylase EglN2 is an estrogen-responsive gene that is highly expressed in ER+ breast cancer, including luminal A and B subtypes, and contributes to breast tumorigenesis (42, 43). We also discovered a novel function of EglN2 as a transcription coactivator that interacts with NRF1 and PGC1α to maintain mitochondrial function during hypoxia (44). FDXR, a mitochondrial flavoprotein, is known to initiate electron transport for cytochrome p450 from NADPH, leading to increased reactive oxygen species (ROS) production (45–47), and acts as a key downstream target gene of the EglN2-NRF1-PGC1α complex, which modulates mitochondrial function and cell proliferation in ER+ breast cancer cells (44). However, the mechanism by which FDXR regulates altered mitochondrial function and supports ER+ breast tumorigenesis is poorly defined.

Here, through an integrative targeted metabolomics assay and gene expression profiling, we showed that FDXR promoted fatty acid oxidation (FAO) by positively regulating CPT1A expression and illustrated that the FDXR-CPT1A-FAO axis was responsible for primary and endocrine-resistant breast cancer cell growth. Furthermore, combined endocrine therapy with FAO inhibitors exerted a synergistic effect on primary and endocrine-resistant breast tumor cell growth. We identified a new FDXR-CPT1A-FAO signaling axis as a promising target for the development of therapies against endocrine resistance in ER+ breast cancer.

## Materials and methods

### Cell culture and reagents

MCF7 and 293T cells were cultured in Dulbecco’s modified Eagle medium (DMEM, Sigma−Aldrich) containing 10% fetal bovine serum (FBS) plus 1% penicillin–streptomycin. T47D cells were cultured in RPMI-1640 (Sigma−Aldrich) containing 10% fetal bovine serum with 1% penicillin–streptomycin. Tamoxifen- or fulvestrant-resistant T47D or MCF7 cells were developed by continuous treatment with tamoxifen (100 nM, > 6 months) or fulvestrant (100 nM, > 4 months), the resistant derivatives were selected when the initially sensitive cells resumed the comparable growth rates to the parental cells, and these cells were cultured in phenol-red free RPMI-1640 medium (Gibco) containing 10% heat-inactivated charcoal-stripped FBS, 1% penicillin–streptomycin and 100 nM 4-OH-tamoxifen or fulvestrant (48, 49). Following viral infection, the cells were maintained in the presence of G418 (100 μg/mL) or puromycin (2 μg/mL) depending on the vector. All cells were maintained in an incubator at 37°C and 5% CO_2_. 4-OH-tamoxifen, fulvestrant and etomoxir were obtained from Sigma−Aldrich. DMNQ and TEMPO were purchased from Selleck.

### Western blotting and antibodies

EBC buffer (50 mM Tris pH 8.0, 120 mM NaCl, 0.5% NP40, 0.1 mM EDTA and 10% glycerol) supplemented with complete protease inhibitor (Roche Applied Biosciences) was used to harvest whole cell lysates. Cell lysate concentrations were measured by the Bradford assay (ThermoFisher Scientific), and equal amounts of proteins were loaded onto an SDS-polyacrylamide gel, separated by electrophoresis and blotted onto a nitrocellulose membrane (Millipore). Rabbit FDXR (15584-1-AP) and CPT1A antibodies (15184-1-AP) were purchased from Proteintech. Mouse Vinculin (V9131) and mouse Flag (F3165) antibodies were purchased from Sigma−Aldrich. Peroxidase-conjugated goat anti-mouse (170-6516) and peroxidase-conjugated goat anti-rabbit (1706515) secondary antibodies were purchased from Bio-Rad.

### Plasmids

Full-length FLAG and HA double-tagged FDXR was amplified by PCR and cloned into the pBABE-puro vector. A KOD-Plus Mutagenesis Kit (SMK-101, TOYOBO) was used to construct FDXR mutants. All plasmids were sequenced to confirm validity.

### Virus production and infection

293T packaging cell lines were used for lentiviral amplification. Lentiviral infection was carried out as previously described (50). Briefly, posttransfection with Lipofectamine 3000, viruses were collected at 48 and 72 h. After being passed through 0.45 μm filters, appropriate amounts of viruses were used to infect target cells in the presence of 8 μg/ml polybrene (Sigma−Aldrich). Subsequently, target cell lines underwent appropriate antibiotic selection.

### siRNAs and lentiviral shRNA vectors

Nontargeting siRNA was obtained from Dharmacon (catalog number: D0012100220). FDXR siRNA (FDXR si434) with the targeting sequence GCUCAGCAGCAUUGGGUAUAA was obtained from Dharmacon.

Lentiviral FDXR shRNAs were obtained from the Broad Institute TRC shRNA library. The target sequences are as follows:

Ctrl shRNA: AACAGTCGCGTTTGC GACTGG;
FDXR (434): GCTCAGCAGCATTGGGTATAA;
FDXR (433): CCATTTCTCCACACAGGAGAA.

### Oxygen consumption rate (OCR) measurement

Extracellular oxygen consumption was determined by measuring the OCRs using a Seahorse XF24 extracellular flux analyzer (Seahorse Bioscience). Approximately 1 × 10^5^ of the indicated cells were seeded into an XF24 cell culture microplate 24 h before the assay. For OCR analysis, baseline mitochondrial respiration was established by recording extracellular oxygen concentrations at several time points. Respiration not linked to mitochondrial ATP synthesis was measured after adding 1 μM oligomycin through an automated injection port of the XF24. Uncoupled respiration measurements were obtained after adding 1 μM FCCP.

### Metabolomics analysis

Intracellular metabolites were prepared and analyzed by LC−MS/MS as described previously (51). Briefly, cells were cultured in 6-well plates (1×10^6^), and the plates were placed on dry ice immediately after the medium was aspirated. Then, 1 ml of 80% methanol/water (precooled at -80°C for at least 1 hour) was added. The plates were transferred to a -80°C freezer for 15 minutes to further inactivate enzymes. Then, the cells were scraped into 80% methanol/water on dry ice and centrifuged at 20,000×*g* for 10 minutes at 4°C. The supernatant was transferred into a new tube and dried with a speed vacuum at room temperature. Finally, the dry pellets were stored at -80°C for further LC−MS/MS analysis (Vanquish, Thermo Fisher Scientific).

### Cell proliferation assays

Tamoxifen- or fulvestrant-resistant T47D or MCF7 cells were plated in triplicate in 96-well plates (3,000 cells/well) in the appropriate growth medium. At the indicated time points, the cells were placed in 90 μl of fresh growth medium supplemented with 10 μl of MTS reagents (Promega) and incubated at 37°C for 2 h. The absorbance value was measured at 490 nm using a 96-well plate reader. The experiments were repeated three times with similar results.

### Colony formation assays

T47D-, MCF7-, tamoxifen- or fulvestrant-resistant cells were trypsinized and plated in triplicate in 6-well dishes (5,000 cells/well). At the indicated time points, the cells were fixed with 4% paraformaldehyde for 15 min at 37°C and then stained with 0.02% crystal violet.

### Anchorage-independent growth assays

Tamoxifen- or fulvestrant-resistant T47D or MCF7 cells were plated at a density of 5,000 cells per ml in complete medium with 0.4% agarose on layers composed of medium with 1% agarose and incubated at 4°C for 10 min. Afterward, the cells were moved to a 37°C incubator. Every 4 days, three drops of complete media were added to the plate. After 2 weeks, the extra liquid on the plate was aspirated, 1 ml of medium was added to each well, and colonies were stained with 100 μg/ml iodonitrotetrazolium chloride solution. The cell culture plates were returned to the incubator overnight, after which the number of foci were counted. The experiments were repeated three times with similar results.

### Bioinformatics analyses of metabolomics data

The metabolomics data were analyzed by R statistical software version 4.1.3. MSEA was performed using MetaboAnalyst (https://www.metaboanalyst.ca/). Differentially regulated metabolites were identified by the edgeR package (v3.36.0) (https://doi.org/10.1093/bioinformatics/btp616) with the thresholds of FDXRsh434/ctrl greater than 1.2 or less than 0.8 following an adjusted p value < 0.05. The heatmaps were drawn with the ComplexHeatmap package (v2.10.0) (https://doi.org/10.1093/bioinformatics/btw313).

### Bioinformatics analyses of the gene expression profile

The gene expression profile was analyzed by R statistical software version 4.1.3. GSEA based on hallmarks was performed using GSEA software (v4.2.3) (https://doi.org/10.1073/pnas.0506580102). Differentially regulated genes were identified by using the limma package (v3.50.3) (https://doi.org/10.1093/nar/gkv007) with an adjusted p value < 0.05. We selected biological process (BP) of GO analysis with the clusterProfiler package (v4.2.2) (https://doi.org/10.1089/omi.2011.0118) in the study. The connection between the significantly enriched fatty acid metabolism GO terms and participating genes was analyzed by the ComplexHeatmap package (v2.10.0).

### Statistical analyses of TCGA and METABRIC datasets

Statistical analyses of these clinical datasets was analyzed by R statistical software version 4.1.3. Survival analyses and Cox proportional hazards models were constructed by using the survival package (v3.5-5). The Kaplan-Meier Curves were drawn with the survminer package (v0.4.9).

### Statistical analysis

The unpaired two-tailed Student’s *t* test was used for experiments comparing two sets of data. The data are presented as the mean ± SEM from three independent experiments. *, **, and *** denote *P* values < 0.05, 0.01, and 0.005, respectively. NS denotes not significant.

### Data availability

The gene expression microarray discussed in this paper have been deposited in NCBI’s Gene Expression Omnibus (GEO) under the accession number GSE217902 and the password is ulgfqumujhkvvqn (https://www.ncbi.nlm.nih.gov/geo/query/acc.cgi?&acc=GSE217902).

The relevant code for R statistical analyses is provided online through https://github.com/whumri-grh/FDXR-Drives-Primary-and-Endocrine-Resistant-1-Tumor-Cell-Growth-in-ER-Breast-Cancer-via-CPT1A-Media.git

## Results

### FDXR is required for fatty acid oxidation and CPT1A expression

**Figure 1:**
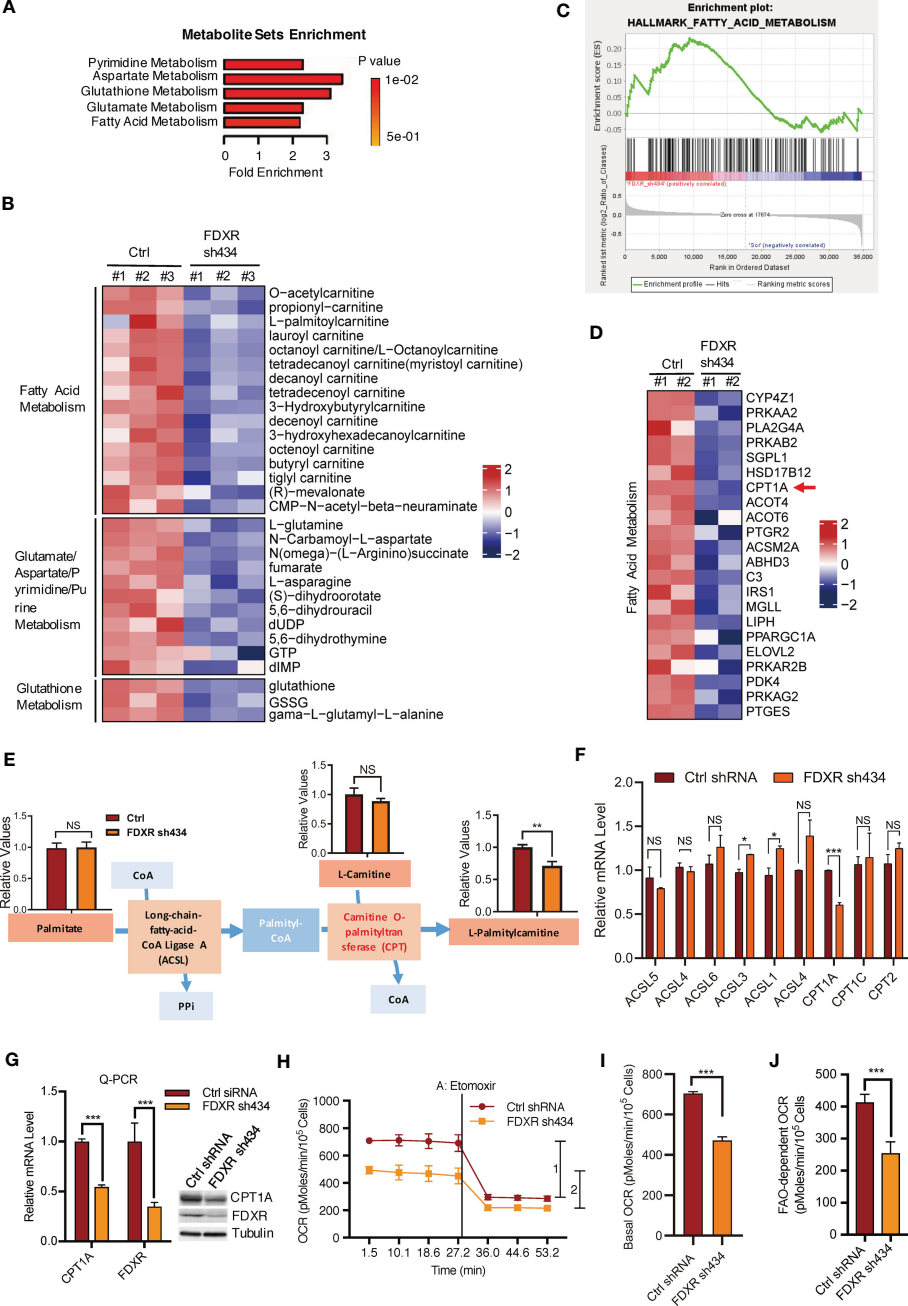
The effect of FDXR on metabolic pathways in ER+ breast cancer cells. **(A)** Metabolite sets enrichment analysis of targeted metabolomics assays in T47D cells infected with lentivirus encoding Ctrl shRNA and FDXR sh434. **(B)** Heatmaps showing the metabolites involved in the indicated metabolic pathways positively regulated by FDXR. **(C)** Gene set enrichment analysis (GSEA) of gene expression profile in T47D cells infected with lentivirus encoding Ctrl shRNA and FDXR sh434. **(D)** Heatmaps showing genes related to fatty acid metabolism positively regulated by FDXR. **(E)** The diagram for FDXR regulation on fatty acid metabolic pathway. **(F)** The relative mRNA levels of fatty acid associated genes under FDXR depletion from gene expression microarray. **(G)** Q-PCR and immunoblots assay to detect FDXR and CPT1A level from T47D cells infected with lentivirus encoding Ctrl shRNA and FDXR sh434. **(H–J)**. Seahorse assays **(H)** and their quantifications of basal OCR **(I)** and indicated 1 and 2 **(J)** for measurement of FAO-dependent OCR under the treatment of an FAO inhibitor etomoxir (40 μM) in T47D cells infected with lentivirus encoding Ctrl shRNA and FDXR sh434. *, **, and *** denote P-value of < 0.05, 0.01, and 0.005, respectively. NS denotes not significant.

We previously reported that FDXR acts as a downstream target gene of the EglN2-NRF1-PGC1α complex to maintain mitochondrial function (44). To explore the mechanism by which FDXR regulates mitochondrial function in breast cancer cells, we performed liquid chromatography (LC) tandem mass spectrometry (MS/MS)-based metabolite profiling in FDXR-knockdown (KD) T47D human ER+ breast cancer cells (**Supplementary Figure 1A**; **Supplementary Table 1**). Among the identified small metabolites, 46 metabolites were significantly decreased and 35 metabolites were significantly increased by FDXR KD (**Supplementary Figure 1B**; **Supplementary Table 2**). Metabolite set enrichment analysis showed that pyrimidine metabolism, aspartate metabolism, glutathione metabolism, glutamate metabolism and fatty acid metabolism were enriched among groups that were positively regulated by FDXR (**Figure 1A**). Among these decreased metabolites, one-third were involved in fatty acid metabolism, and almost all of them belonged to acyl-carnitine (**Figure 1B**; **Supplementary Table 3**). Moreover, gene expression profiling of FDXR KD T47D cells showed that a set of metabolism-related genes were positively regulated by FDXR (**Supplementary Figure 1C**; **Supplementary Table 4**). Gene set enrichment analysis (GSEA) showed that fatty acid metabolism was enriched in groups that were positively regulated by FDXR (**Figure 1C**). Specifically, we found that CPT1A, which catalyzes acyl-CoA to acyl-carnitine to allow mitochondrial uptake of long-chain fatty acids and is a key rate-limiting enzyme for fatty acid oxidation (**Supplementary Figure 1D**) (52), was significantly decreased by FDXR KD in T47D lines (**Figure 1D**; **Supplementary Table 5**). Therefore, we hypothesized that the decrease in CPT1A is the main reason for the reduction in acyl-carnitine in FDXR-depleted cells.

To test this hypothesis, we specifically showed the metabolite levels related to the synthesis of palmitylcarnitine from palmitate (**Figure 1E**). Through LC-MS/MS, we confirmed that FDXR depletion decreased palmitylcarnitine, which is generated through CPT1-mediated catalysis of palmityl-CoA, but had no effect on palmitate or carnitine (**Figure 1E**). The gene expression profile showed that among the enzymes involved in the formation of palmitylcarnitine, only CPT1A was reduced by FDXR depletion (**Figure 1F**). Furthermore, Q-PCR and western blotting assays confirmed that FDXR depletion inhibited CPT1A expression (**Figure 1G**). The integrative analyses of the targeted metabolomics assay and gene expression profiling suggest that FDXR KD decreased acyl-carnitine by downregulating CPT1A expression. Since CPT1A is a key enzyme for fatty acid oxidation, we hypothesized that FDXR may consequently regulate fatty acid oxidation. We measured the oxygen consumption rate (OCR) with an XF-24 extracellular flux analyzer and found that FDXR KD inhibited basal levels of mitochondrial respiration. The decreased amount of OCR caused by etomoxir represents the amount of OCR derived from FAO pathway, therefore, we treated cells with etomoxir, an inhibitor of FAO that targets CPT1 used to measure FAO-dependent OCR in cells, and found that FDXR KD-mediated inhibition of OCR was largely mediated by FAO (**Figures 1H–J**). Collectively, these results suggest that FDXR positively regulates CPT1A expression and fatty acid oxidation.

### FDXR regulates fatty acid oxidation and tumor cell growth through CPT1A

**Figure 2:**
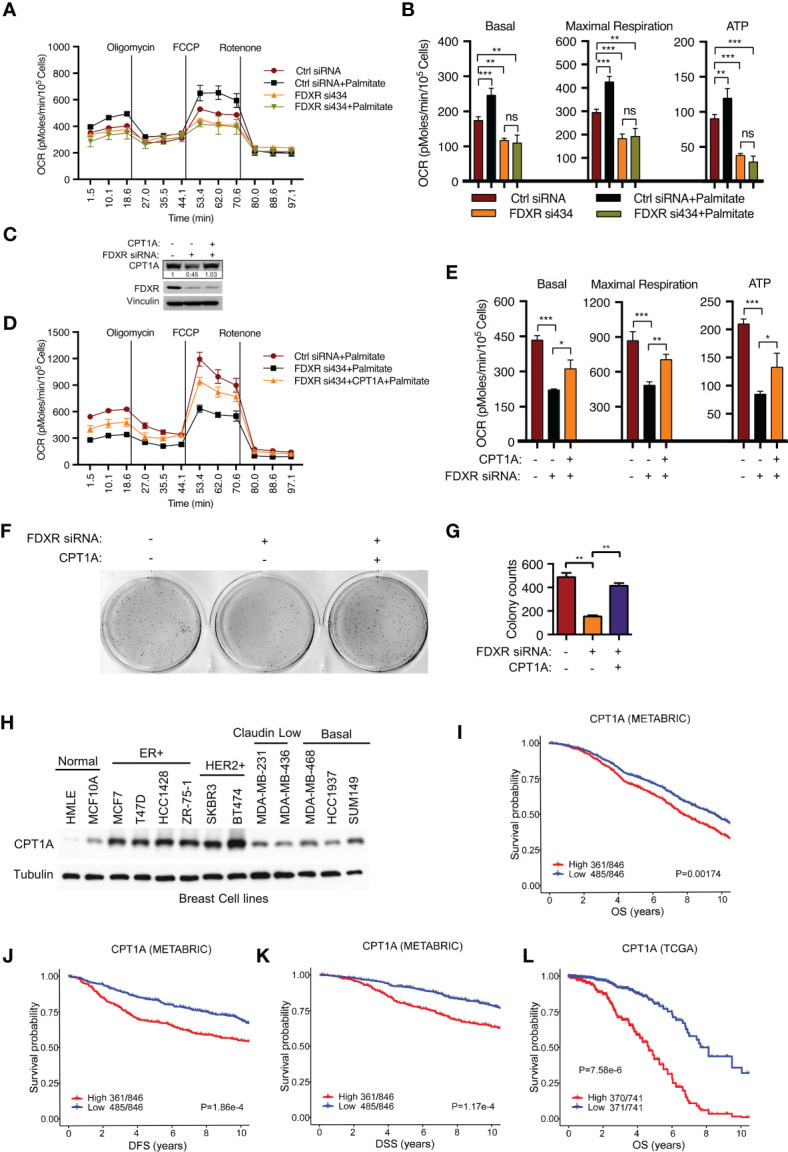
FDXR regulates fatty acid oxidation and tumor cell growth through CPT1A in ER+ breast cancer cells. **(A, B)** Seahorse assays **(A)** and their quantifications of basal, maximal respiration, or ATP production **(B)** from T47D cells transfected with Ctrl siRNA and FDXR si434. **(C–G)**. Immunoblots **(C)**, Seahorse assays **(D)** and their quantifications of basal, maximal respiration, or ATP production **(E)**, soft agar assays **(F)** and their quantifications **(G)** from T47D cells infected with vector (control) or CPT1A followed by transfection with Ctrl siRNA and FDXR si434. **(H)** Immunoblots of the breast cell lines as indicated. **(I–L)**. The Kaplan–Meier curves of overall survival (OS) **(I, L)**, disease free survival (DFS) **(J)**, and disease special survival (DSS) **(K)** based on CPT1A expression in ERα-positive patients from METABRIC **(I–K)** and TCGA **(L)** cohorts. Patients were rank-ordered and divided into two equal groups (low in blue and high in red), using the CPT1A gene expression levels. *, **, and *** denote P-value of < 0.05, 0.01, and 0.005, respectively. ns denotes not significant.

To verify whether FDXR KD-mediated inhibition of fatty acid oxidation occurs through the downregulation of CPT1A, we further examined FAO-driven OCR by adding exogenous palmitate, which served as the sole carbon source driving OXPHOS. We found that FDXR KD abrogated palmitate-derived OCR, decreased basal and maximal respiration, and inhibited ATP production (**Figures 2A, B**). Then, we reconstituted CPT1A in FDXR KD cells and found that CPT1A could partially rescue the decrease in OCR caused by FDXR KD (**Figures 2C–E**), indicating that FDXR regulates FAO at least partially through CPT1A. Our data showed that depletion of FDXR inhibited tumor cell growth in T47D and MCF7 breast cancer cell lines (44) (**Supplementary Figures 2A–D**), and this phenotype could be at least partially rescued by the reconstitution of CPT1A expression (**Figures 2F, G**). Thus, our data indicate that FDXR regulates tumor cell growth through CPT1A. Similar to FDXR, as we previously reported (44), CPT1A was upregulated in breast cancer cell lines compared to the human mammary epithelial cell lines HMLE and MCF10A (**Figure 2H**), and higher expression of CPT1A was associated with decreases in overall survival (OS) (**Figures 2I, L**), disease-free survival (DFS) (**Figure 2J**) and disease-specific survival (DSS) (**Figure 2K**) in the METABRIC and TCGA ER+ breast patient datasets (**Supplementary Tables 6**, **7**). These data reveal FDXR-CPT1A-FAO axis as a potential target for breast cancer.

### FDXR is required for endocrine-resistant ER+ breast tumor growth

**Figure 3:**
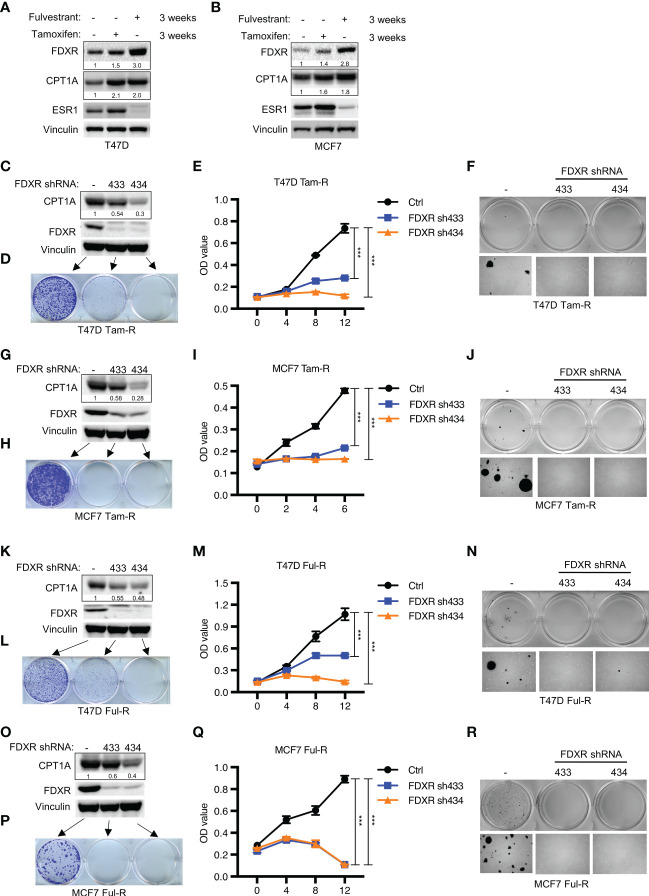
The effect of FDXR depletion on endocrine resistant ER+ breast cancer cell growth. **(A, B)** Immunoblot assays of T47D **(A)** or MCF7 **(B)** with three weeks of treatment with fulvestrant (0.1 μM) or tamoxifen (0.1 μM). **(C–R)** Immunoblot assays **(C, G, K, O)**, 2D colony formation **(D, H, L, P)**, cell proliferation **(E, I, M, Q)** and soft agar assay **(F, J, N, R)** from T47D or MCF7 tamoxifen-resistant (T47D Tam-R, MCF7 Tam-R) or fulvestrant-resistant (T47D Ful-R, MCF7 Ful-R) cells infected with lentivirus encoding Ctrl shRNA and FDXR shRNA (433 or 434). *** denote P-value of < 0.005.

Endocrine therapy is the first-line clinical treatment for estrogen receptor-positive (ER+) breast cancer, but long-term hormone therapy, such as tamoxifen and fulvestrant, can lead to the development of endocrine resistance (53). Increasing evidence indicates that endocrine-resistant tumor cells exhibit a high mitochondrial OXPHOS status (54). Therefore, we examined whether FDXR-CPT1A-FAO axis-driven OXPHOS was responsible for endocrine resistance in breast cancer. According to previous reports (48, 49, 55), T47D and MCF7 cells were treated with 0.1 μM tamoxifen or fulvestrant for three weeks to induce drug resistance, and we found that long-term treatment with tamoxifen or fulvestrant increased the expression levels of FDXR and CPT1A (**Figures 3A, B**). Furthermore, western blotting, cell proliferation assays and anchorage-independent growth assays showed that FDXR KD in tamoxifen- or fulvestrant-resistant T47D and MCF7 cell lines inhibited CPT1A expression and decreased tumor cell growth (**Figures 3C–R**; **Supplementary Figures 3A–D**). As the endocrine-resistant cells were cultured in the presence of 100 nM tamoxifen or fulvestrant, and FDXR depletion along with this constant endocrine treatment in those cells blocked cell growth, implying that FDXR may sensitize these endocrine-resistant cells back to endocrine treatment and is essential for endocrine-resistant cell growth.

### Endocrine-resistant ER+ breast cancer cells are highly dependent on fatty acid oxidation compared with primary cells

**Figure 4:**
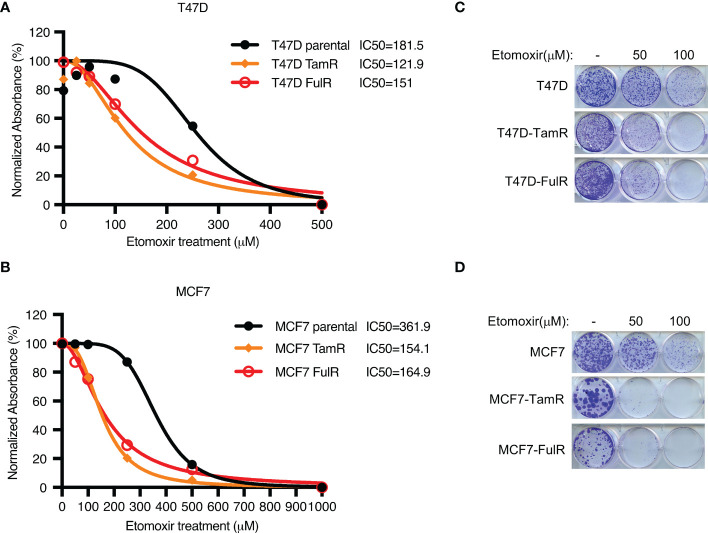
The effect of CPT1 inhibitor etomoxir on primary and endocrine resistant ER+ breast cancer cell growth. **(A–D)** Cell proliferation **(A, B)** and 2D colony formation **(C, D)** from T47D or MCF7 cells with or without the indicated doses of etomoxir treatment for 4 days and 10 days, respectively.

Given that both FDXR and CPT1A were upregulated by long-term treatment with tamoxifen or fulvestrant, we hypothesized that endocrine-resistant cells may rely more on the FDXR-CPT1A-FAO axis to survive the harsh environment than primary cells. To test this hypothesis, we performed MTT assays on primary and endocrine-resistant ER+ breast cancer cells treated with various concentrations of etomoxir. Our data showed that etomoxir inhibited the proliferation of primary and endocrine-resistant T47D and MCF7 cells, but endocrine-resistant breast cancer cells showed much lower IC50 values for etomoxir treatment (**Figures 4A, B**; **Supplementary Figures 4A, B**). In addition, 2D colony formation assays showed that endocrine-resistant breast cancer cells were more sensitive to etomoxir treatment than primary breast cancer cells (**Figures 4C, D**), indicating that endocrine-resistant breast cancer cells are more dependent on fatty acid oxidation. These data suggest that fatty acid oxidation is required for both primary and endocrine-resistant ER+ breast cancer cell proliferation, and endocrine-resistant cells rely highly on fatty acid oxidation compared with primary cells.

### Combining a CPT1 inhibitor with fulvestrant treatment synergistically reduces primary and endocrine-resistant ER+ breast cancer cell growth

**Figure 5:**
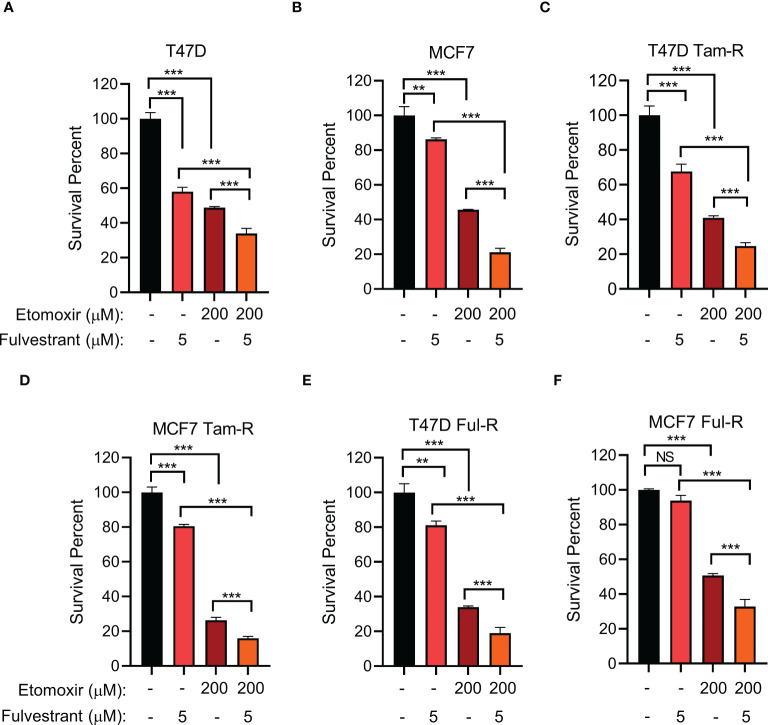
The effect of combined CPT1 inhibitor and fulvestrant treatment on primary and endocrine resistant ER+ breast tumor cell growth. **(A–F)** Cell survival analysis of the indicated T47D or MCF7 cell lines under the indicated treatments for 4 days. **, and *** denote P-value of < 0.05, 0.01, and 0.005, respectively. NS denotes not significant.

Given that long-term tamoxifen or fulvestrant treatment increased CPT1A levels, we hypothesized that inhibiting CPT1A might synergize with endocrine therapy to exert enhanced therapeutic efficacy. We firstly examined the proper concentrations of tamoxifen/fulvestrant and CPT1 inhibitor etomoxir for their combinatory treatment by using different doses, we found that combining fulvestrant with etomoxir showed synergistic effect on inhibiting endocrine-resistant cancer cell growth in a dose-dependent manner (**Supplementary Figures 5A–F**). Moreover, our data showed that primary cells are indeed more sensitive than endocrine-resistant cells to endocrine treatment (**Supplementary Figures 5G, H**). Further, we found that treatment with either fulvestrant or etomoxir alone led to differential inhibition of cell proliferation in both primary and endocrine-resistant ER+ breast cancer, and combining etomoxir with fulvestrant resulted in a synergistic decrease in cell proliferation (**Figures 5A–F**). However, combining etomoxir with tamoxifen showed no synergistic effect on cell growth (**Supplementary Figures 5A, B**; **Supplementary Figure 6**) (56). Thus, our data reveal that the CPT1 inhibitor etomoxir restores the sensitivity of endocrine-resistant breast cancer cells to fulvestrant and that combining etomoxir with fulvestrant synergistically reduces breast cancer cell growth.

## Discussion

Endocrine therapies are the first-line treatment for early-stage ER+ breast cancers, but many patients relapse and develop drug resistance (53, 57). Understanding the factors and pathways that drive drug resistance has allowed the development of subsequent therapies and helped guide decision-making to maximize efficacious and successful treatment of cancers (58). Accumulating evidence has suggested that metabolic reprogramming is associated with drug resistance (59–61). Investigating metabolic alterations and therefore exploiting metabolic vulnerabilities in cancers are critical for precision medicine (62). Mitochondrial respiration is increased in response to endocrine resistance in ER+ breast cancer; however, the mechanism is not well understood (63). Previously, we showed that the EglN2-NRF1-PGC1α axis regulates mitochondrial function by promoting FDXR expression, but it is unclear how FDXR regulates mitochondrial function. Through an integrative targeted metabolomics assay and gene expression profiling approach, we found that FDXR was essential for fatty acid oxidation and mitochondrial respiration through the positive regulation of CPT1A. Furthermore, we found that endocrine-resistant breast cancer cells highly depend on the fatty acid oxidation pathway compared with primary cells, and the combination of endocrine therapy with an FAO inhibitor synergistically inhibits primary and endocrine-resistant breast cancer cell growth. Thus, we reveal an important mechanism of mitochondrial adaptation to endocrine treatment and provide a new therapeutic strategy by combining endocrine therapy with FAO inhibitors.

We found that FDXR correlated with mitochondrial OXPHOS genes in the ER+ breast cancer patients, while CPT1A did not have this correlation. Also, FDXR and CPT1A did not correlated in the ER+ breast cancer patients (**Supplementary Figures 7A–C**; **Supplementary Table 8**), the reason could be that both CPT1A and mitochondrial OXPHOS genes were downstream genes regulated by FDXR, and also other factors may co-regulate CPT1A expression. FDXR, which is a mitochondrial flavoprotein that initiates electron transport from NADPH, positively regulates ROS production (47) (**Supplementary Figure 8**). But whether this process contributes to mitochondrial OXPHOS and endocrine resistance requires further investigation. Also, we have been focusing on the regulatory mechanism of how FDXR regulates mitochondrial oxidative phosphorylation in this study, whether FDXR regulates the extracellular acidification rate (ECAR) and modulates oxidization of other primary mitochondrial fuels glucose and glutamine needs future study. Besides, it takes much longer time for obtaining the endocrine-resistant tumor tissues from breast cancer patients, future study will be needed to validate the roles of FDXR and CPT1A in endocrine resistance *in vivo* by patient-derived xenograft (PDX) models.

Lipid metabolism has been recognized as essential for tumor cell growth and progression (64, 65), and it has emerged as a promising target for many cancers (66, 67). Accumulating evidence suggests that alterations in lipid metabolism mediate the development of acquired drug resistance in various types of cancers, including breast cancer (60, 61). In this study, through combining different datasets derived from two separate cohorts of ER+ breast cancer patients, we show that higher expression of CPT1A is associated with worse prognosis. Further, we also find that the endocrine-resistant cell lines are highly sensitive to CPT1A inhibitor compared with primary cell lines, and endocrine treatment leads to upregulation of CPT1A, implying that cancer cells develop CPT1A-mediated pathway for cellular adaptation to the new environment. Therefore, CPT1A could be used as a biomarker and tested at baseline and after recurrence. As such, targeting CPT1A has a potential to be used in combination therapy with endocrine treatment. Our data have showed that endocrine resistant cells highly depend on FAO compared with primary cells, and inhibition of FAO with etomoxir significantly inhibits endocrine resistant cell growth. It is well-known that metabolic reprogramming plays an important role in development of drug resistance (59), but whether cell metabolisms can be reprogrammed towards glycolysis or glutamine following endocrine or etomoxir treatment awaits future investigation. It has reported that inhibition of glycolysis results in upregulation of FAO (68), but whether inhibition of FAO leads to metabolic reprogramming towards glycolysis or glutamine needs future study. These studies may provide additional combination therapies for breast cancer.

## Data availability statement

The datasets presented in this study can be found in online repositories. The names of the repository/repositories and accession number(s) can be found in the article/**Supplementary Material**.

## Author contributions

QZ and JZ conceived and supervised the project. JZ and CY analyzed the data. JZ and CY performed most of experiments. RG and CG performed bioinformatics analyses. KH and MC performed plasmid construction. XL helped with metabolomics assays. QZ, JZ, and CY wrote the paper with critical comments from all authors. All authors contributed to the article and approved the submitted version.

## Supplementary material

Supplementary Figure 1 Integrative analyses of the targeted metabolomics and gene expression prolife reveal FDXR-mediated gene regulation of cell metabolism in ER+ breast cancer. **(A)** Heatmap analysis of the targeted metabolomics assays from T47D cells infected with lentivirus encoding Ctrl shRNA and FDXR sh434. **(B)** Heatmaps showing differentially regulated metabolites affected by FDXR in T47D cells infected with lentivirus harboring either Ctrl shRNA or FDXR sh434. **(C)** Heatmaps showing metabolism-related genes positively regulated by FDXR from T47D cells infected with lentivirus encoding Ctrl shRNA and FDXR sh434. **(D)** Schematic diagram showing CPT1-mediated regulation of fatty acid oxidation pathway to feed TCA cycle for mitochondrial oxidative phosphorylation.

Supplementary Figure 2 FDXR is essential for MCF7 cells proliferation. **(A–D)** Immunoblot assays **(A)**, cell proliferation **(B)**, soft agar assay **(C)** and quantification **(D)** for MCF7 cells infected with lentivirus encoding Ctrl shRNA and FDXR shRNA (433 or 434). *** denote P-value of < 0.005.

Supplementary Figure 3 FDXR is required for the endocrine resistant ER+ breast cancer cell growth. **(A–D)** Quantification of soft agar assays described in **Figures 3D, H, L, P**, respectively. *** denote P-value of < 0.005.

Supplementary Figure 4 The effect of CPT1 inhibitor etomoxir on primary and endocrine resistant ER+ breast cancer cell growth. **(A, B)** Cell proliferation of primary or endocrine resistant T47D or MCF7 cells with or without the indicated doses of etomoxir treatment for 4 days. *, **, and *** denote P-value of < 0.05, 0.01, and 0.005, respectively. NS denotes not significant.

Supplementary Figure 5 The effect of CPT1 inhibitor etomoxir on primary and endocrine resistant ER+ breast cancer cell growth. **(A–F)** Cell survival analysis of the indicated primary or endocrine resistant T47D or MCF7 cell lines under the indicated treatments for 4 days. **(G, H)** MTS assays of primary or endocrine resistant T47D or MCF7 cells with or without tamoxifen (5 μM) or fulvestrant (5 μM) treatment for 4 days. *, **, and *** denote P-value of < 0.05, 0.01, and 0.005, respectively. NS denotes not significant.

Supplementary Figure 6 The effect of combined etomoxir and tamoxifen treatment on tamoxifen-resistant ER+ breast cells. Cell survival analysis from tamoxifen-resistant T47D cells with the indicated treatments with etomoxir (200 μM) and tamoxifen (5 μM) for 4 days. *, **, and *** denote P-value of < 0.05, 0.01, and 0.005, respectively. NS denotes not significant.

Supplementary Figure 7 Correlation of FDXR with OXPHOS-related gene expression in ER+ patients. **(A, B)** Correlation analysis of FDXR or CPT1A with OXPHOS-related parameters in the ER+ cohort of patients. **(C)** Correlation between FDXR and CPT1A in the ER+ cohort of patients.

Supplementary Figure 8 Silencing of FDXR reduces cellular ROS production. ROS level analysis by using DCFH-DA from T47D cells transfected with FDXR siRNA. * denote P-value of < 0.05.
