# Supplementary figures and images for: FDXR drives primary and endocrine-resistant tumor cell growth in ER+ breast cancer via CPT1A-mediated fatty acid oxidation
Source: Front Oncol. 2023 May 3;13:1105117. doi: 10.3389/fonc.2023.1105117 (PMC10189134; doi:10.3389/fonc.2023.1105117)

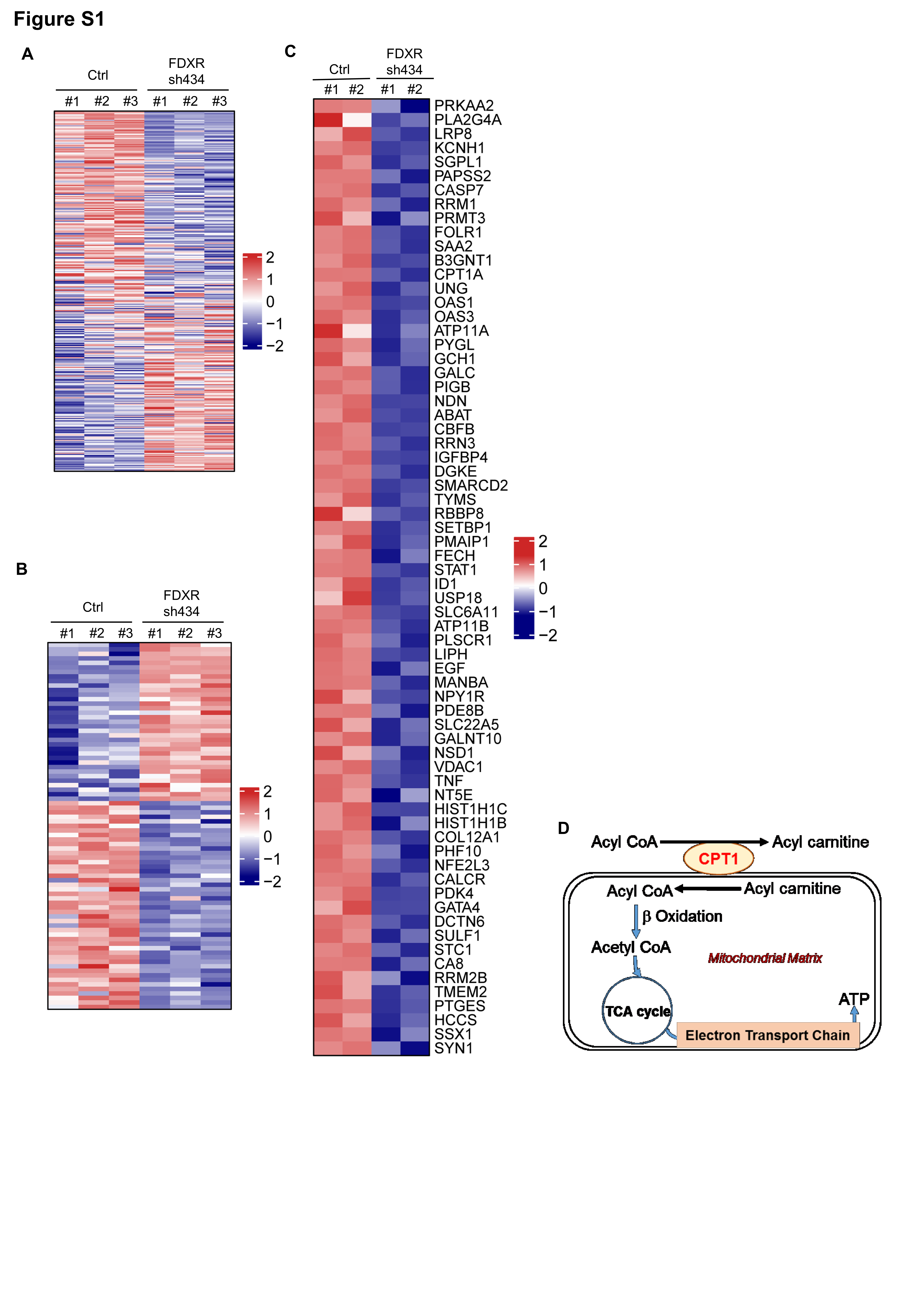

Supplement: Supplementary Figure 1 — Integrative analyses of the targeted metabolomics and gene expression prolife reveal FDXR-mediated gene regulation of cell metabolism in ER+ breast cancer. (A) Heatmap analysis of the targeted metabolomics assays from T47D cells infected with lentivirus encoding Ctrl shRNA and FDXR sh434. (B) Heatmaps showing differentially regulated metabolites affected by FDXR in T47D cells infected with lentivirus harboring either Ctrl shRNA or FDXR sh434. (C) Heatmaps showing metabolism-related genes positively regulated by FDXR from T47D cells infected with lentivirus encoding Ctrl shRNA and FDXR sh434. (D) Schematic diagram showing CPT1-mediated regulation of fatty acid oxidation pathway to feed TCA cycle for mitochondrial oxidative phosphorylation. [file Image_1.tif]

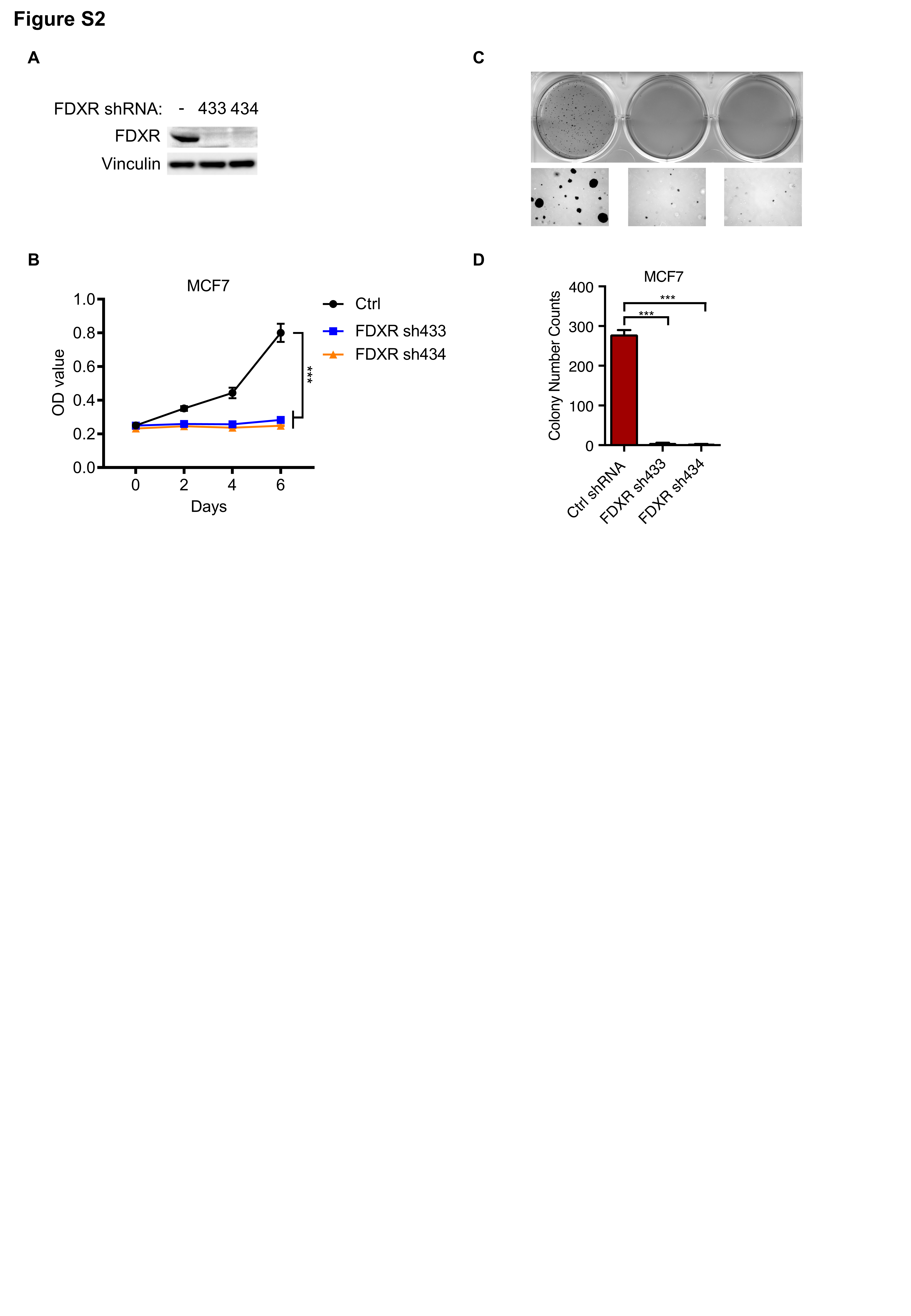

Supplement: Supplementary Figure 2 — FDXR is essential for MCF7 cells proliferation. (A–D) Immunoblot assays (A), cell proliferation (B), soft agar assay (C) and quantification (D) for MCF7 cells infected with lentivirus encoding Ctrl shRNA and FDXR shRNA (433 or 434). *** denote P-value of < 0.005. [file Image_2.tif]

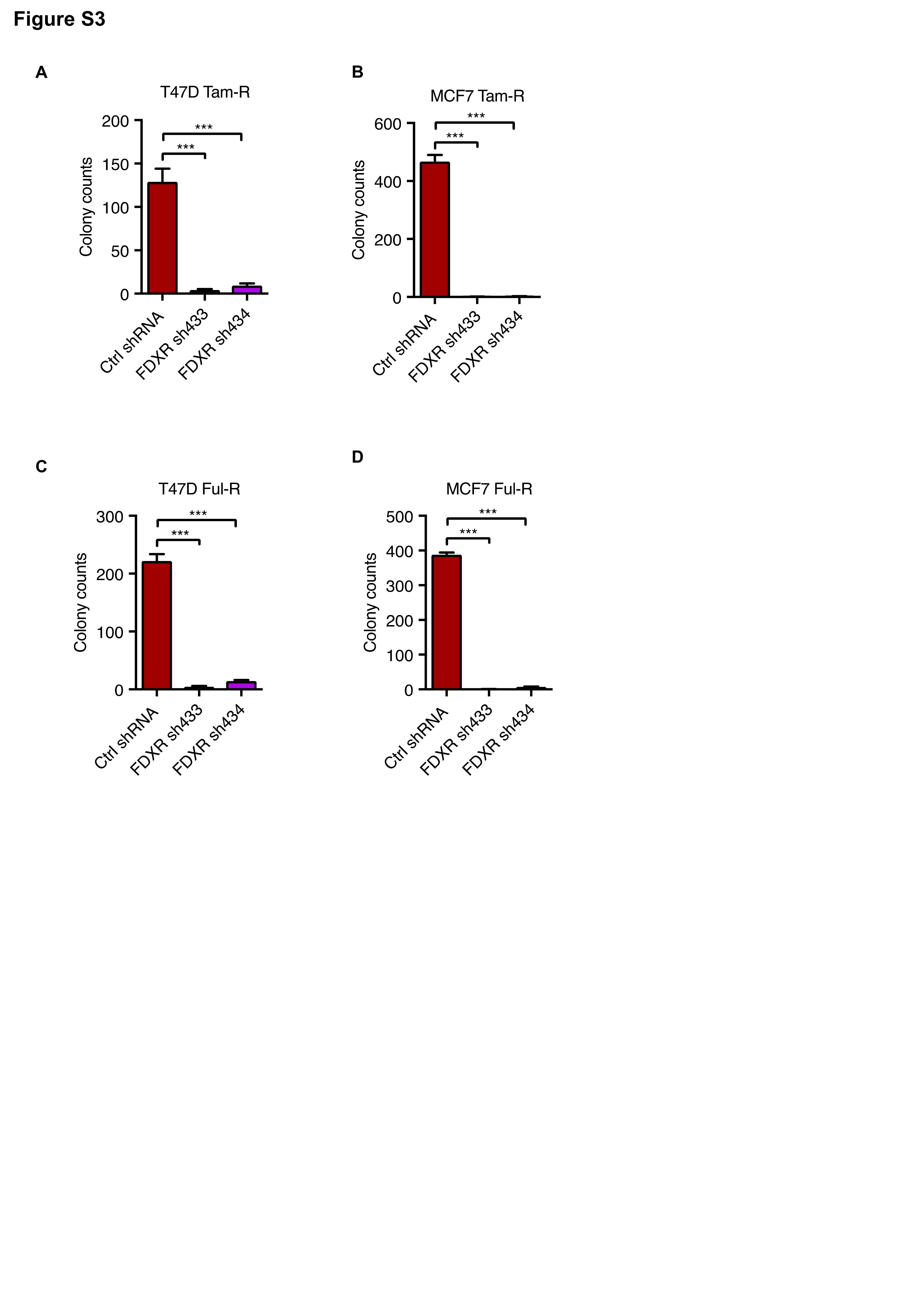

Supplement: Supplementary Figure 3 — FDXR is required for the endocrine resistant ER+ breast cancer cell growth. (A–D) Quantification of soft agar assays described in Figures 3D, H, L, P , respectively. *** denote P-value of < 0.005. [file Image_3.tif]

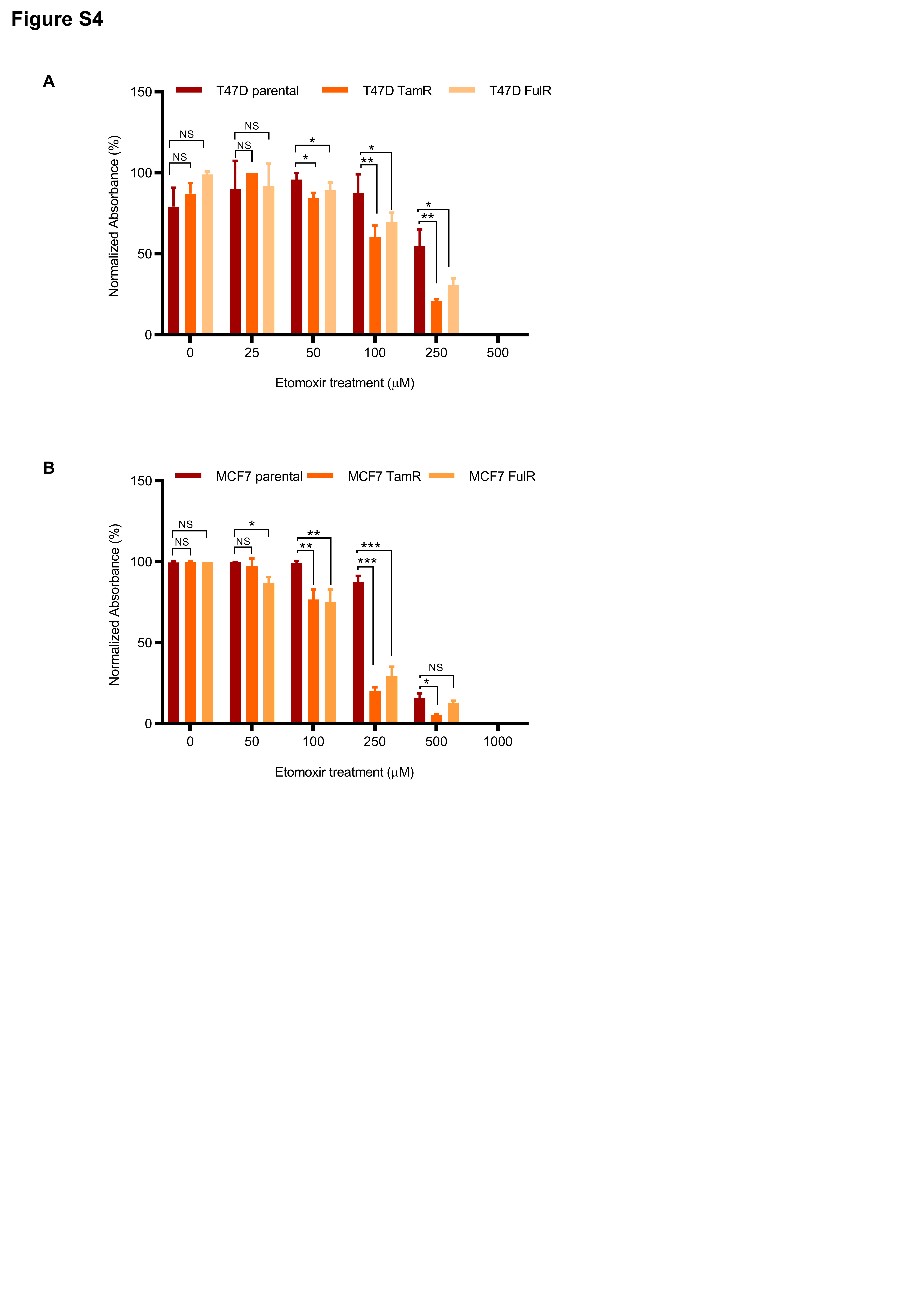

Supplement: Supplementary Figure 4 — The effect of CPT1 inhibitor etomoxir on primary and endocrine resistant ER+ breast cancer cell growth. (A, B) Cell proliferation of primary or endocrine resistant T47D or MCF7 cells with or without the indicated doses of etomoxir treatment for 4 days. *, **, and *** denote P-value of < 0.05, 0.01, and 0.005, respectively. NS denotes not significant. [file Image_4.tif]

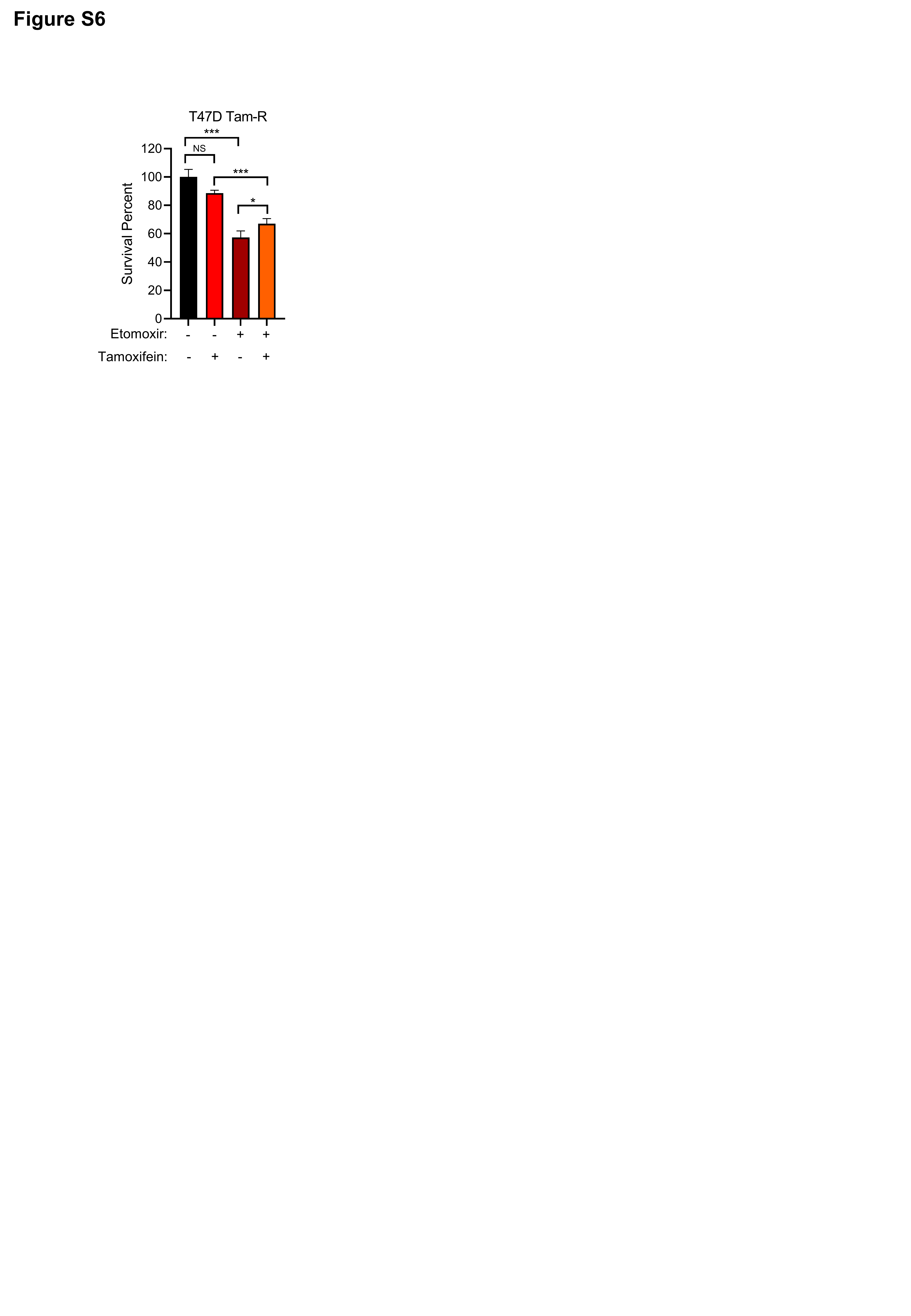

Supplement: Supplementary Figure 5 — The effect of CPT1 inhibitor etomoxir on primary and endocrine resistant ER+ breast cancer cell growth. (A–F) Cell survival analysis of the indicated primary or endocrine resistant T47D or MCF7 cell lines under the indicated treatments for 4 days. (G, H) MTS assays of primary or endocrine resistant T47D or MCF7 cells with or without tamoxifen (5 μM) or fulvestrant (5 μM) treatment for 4 days. *, **, and *** denote P-value of < 0.05, 0.01, and 0.005, respectively. NS denotes not significant. [file Image_5.tif]

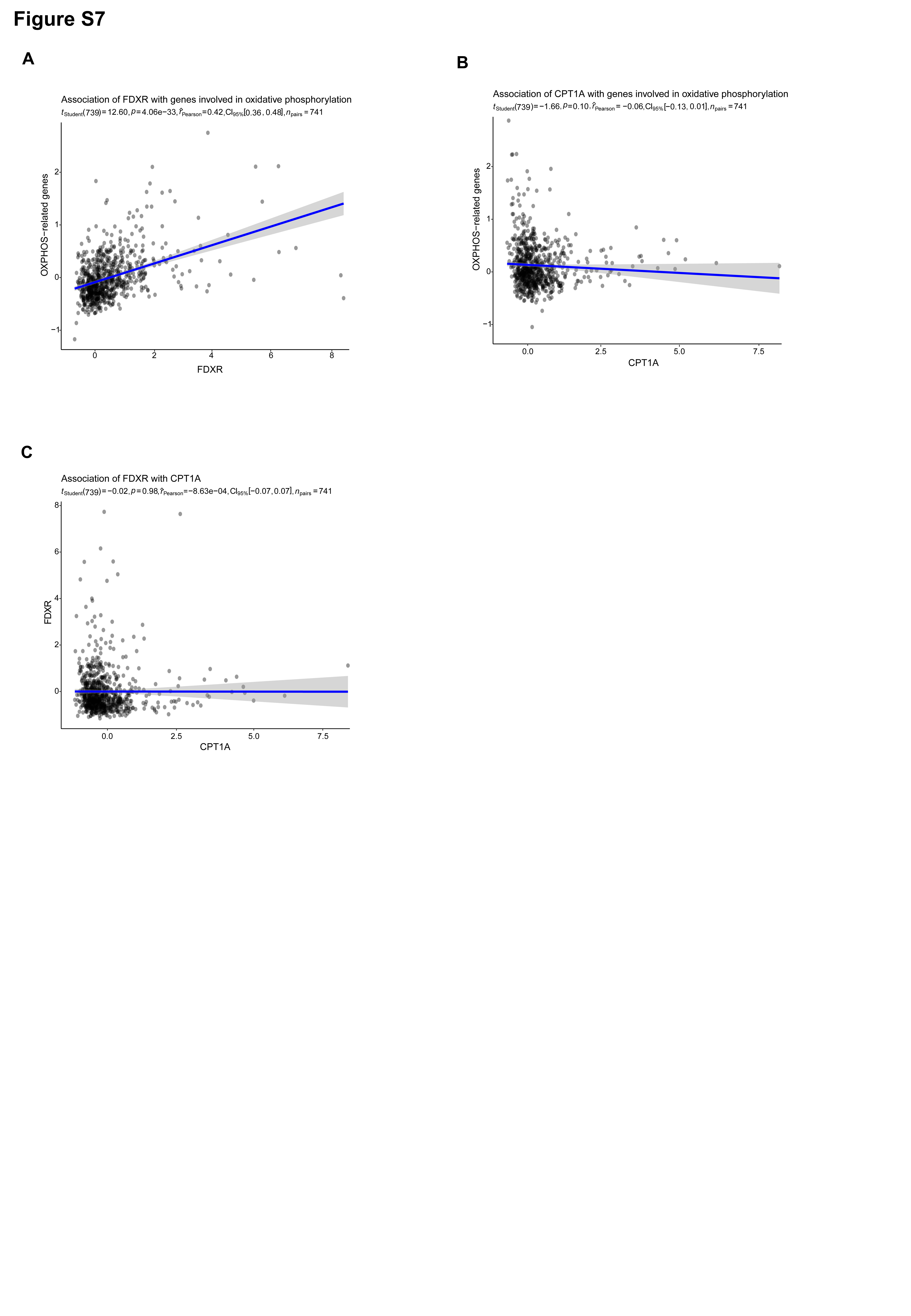

Supplement: Supplementary Figure 6 — The effect of combined etomoxir and tamoxifen treatment on tamoxifen-resistant ER+ breast cells. Cell survival analysis from tamoxifen-resistant T47D cells with the indicated treatments with etomoxir (200 μM) and tamoxifen (5 μM) for 4 days. *, **, and *** denote P-value of < 0.05, 0.01, and 0.005, respectively. NS denotes not significant. [file Image_6.tif]

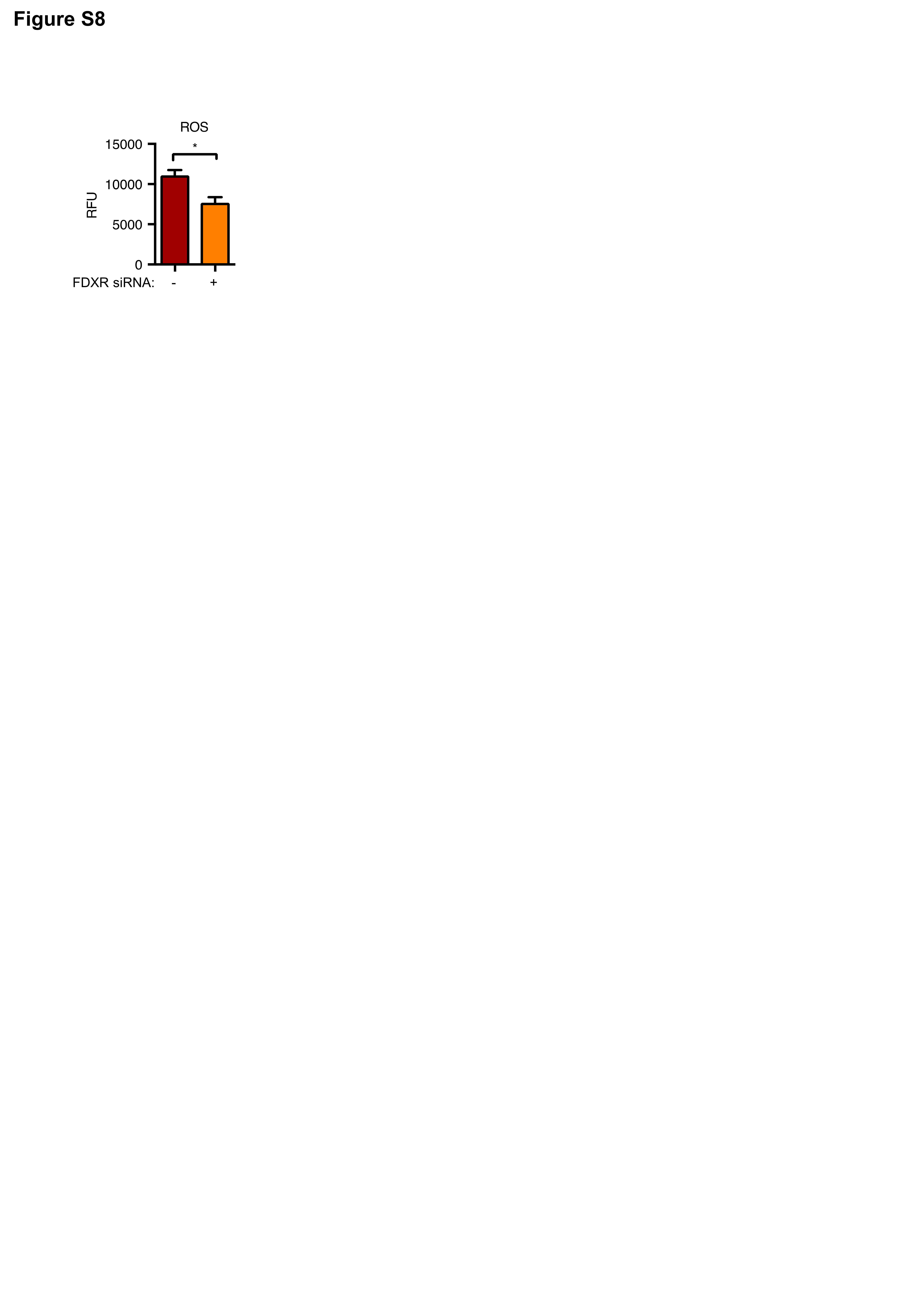

Supplement: Supplementary Figure 7 — Correlation of FDXR with OXPHOS-related gene expression in ER+ patients. (A, B) Correlation analysis of FDXR or CPT1A with OXPHOS-related parameters in the ER+ cohort of patients. (C) Correlation between FDXR and CPT1A in the ER+ cohort of patients. [file Image_7.tif]

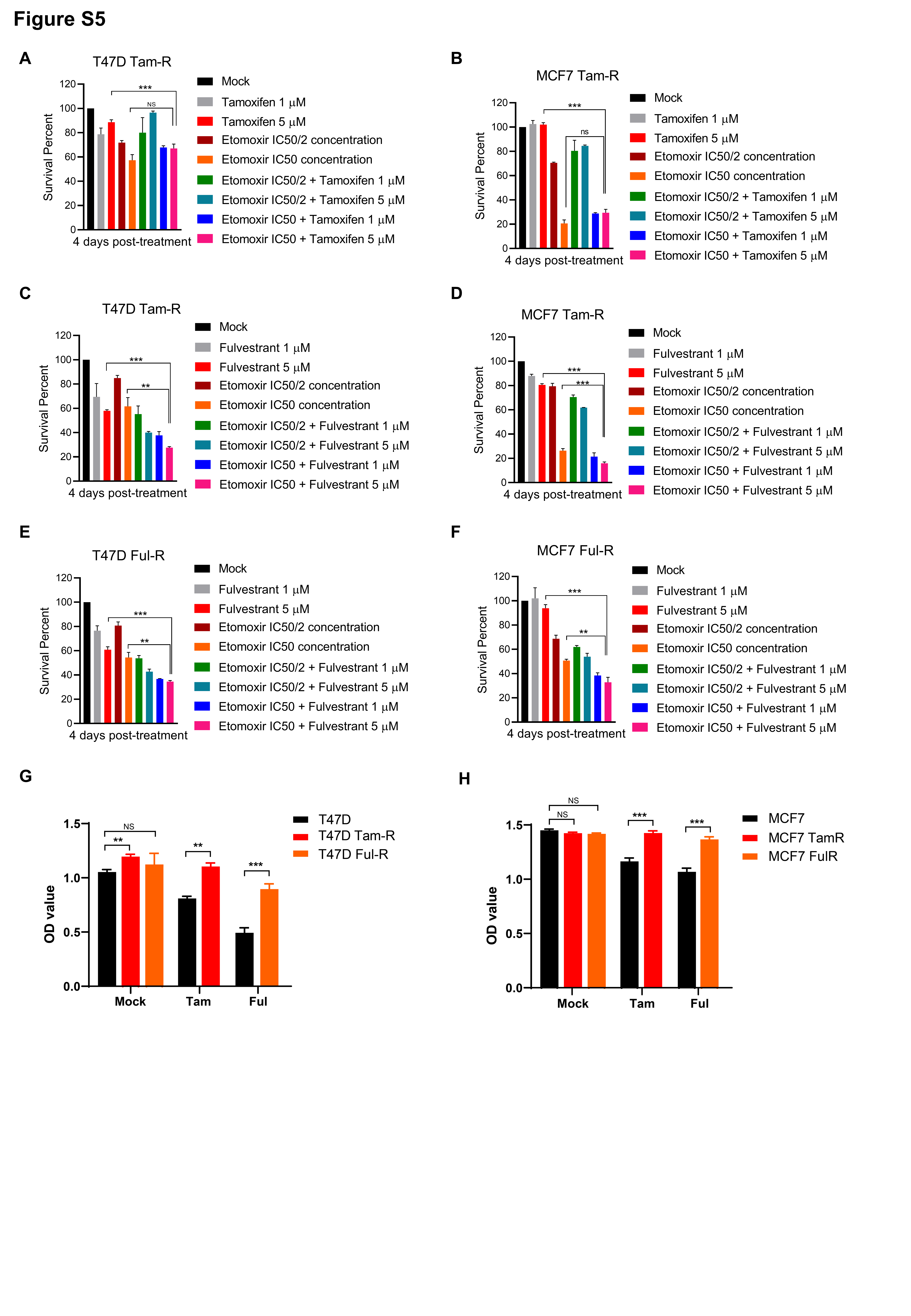

Supplement: Supplementary Figure 8 — Silencing of FDXR reduces cellular ROS production. ROS level analysis by using DCFH-DA from T47D cells transfected with FDXR siRNA. * denote P-value of < 0.05. [file Image_8.tif]
